# Effects of Transglutaminase-Induced β-Conglycinin Gels on Intestinal Morphology and Intestinal Flora in Mice at Different High-Intensity Ultrasound Pretreatment Time

**DOI:** 10.3390/foods13142192

**Published:** 2024-07-11

**Authors:** Jixin Zhang, Lan Zhang, Huiqing Xu, Jun Wang

**Affiliations:** College of Tourism and Culinary Institute, Yangzhou University, Yangzhou 225127, China; xxwyzjx@163.com (J.Z.); mx120211221@stu.yzu.edu.cn (L.Z.); 007232@yzu.edu.cn (J.W.)

**Keywords:** ultrasound pretreatment time, β-conglycinin, transglutaminase-induced gel, intestinal morphology, intestinal flora

## Abstract

TGase-7S gels prepared after different HIU pretreatment times were used to intervene in healthy mice to analyze their effects on growth characteristics and intestinal morphology, and 16S rRNA high-throughput sequencing was applied to fecal samples to investigate the effects of the gel on the structure and diversity of intestinal flora in mice. The results showed that the intestinal tissues of mice in different treatment groups showed better integrity, and the intake of gel increased the length of small intestinal villi in mice, among which the 30-gel group had the highest value of villi length (599.27 ± 44.28) μm (*p* < 0.05) and showed the neatest and tightest arrangement, indicating that the intake of gel did not have adverse effects on the intestinal tract. The effect of gel ingestion on the diversity of the intestinal microbial community structure was more significant, positively promoting the growth of beneficial bacteria such as *Desferriobacterium*, *Synechococcus*, and *Bifidobacterium*. In addition, the ingestion of the gel improved the intestinal health of mice by altering the physiological functions of the intestinal flora and modulating their participation in various metabolic pathways. The above findings provide some theoretical value for the safety of 7S gel in food applications.

## 1. Introduction

In a previous study, we investigated the effect of different high-intensity ultrasound pretreatment (HIU) times on the properties of β-conglycinin (7S) gels induced by the transglutaminase (TGase) method. This provides theoretical and technical support for the development of high-quality 7S gel products and promotes new applications of 7S in the food processing industry [1]. However, do TGase-induced 7S gels prepared at different HIU pretreatment times have an effect on organismal health? What is the metabolic mechanism? It is not clear so far.

Gut flora plays an important role in host health and disease [2], and alterations in the structure of the gut microbial community are closely linked to complex metabolic interactions, energy absorption, and consumption in the host organism [3,4,5]. In addition, the products of protein digestion and absorption can influence the body’s metabolites and cause dramatic changes in the structure of the gut microbial community. Zhu et al. [6], in comparing the effects of pork protein, fish protein, beef protein and soy protein on the structure of the intestinal microbial community, also found that there was a great difference in the alteration of the intestinal flora in terms of the number and type of dietary proteins. Fu et al. [7] demonstrated that the intake of millet proteins influenced the structure of the intestinal microbial community, allowing the activation of the PI3K/AKT pathway to maximize its hypoglycemic effect.

On the other hand, transglutaminase TGase-mediated enzymatic reactions induce the formation of ε-(γ-glutamyl)lysine isopeptide bonds (G-L isopeptide bonds) between the ε-amino group of lysine and the γ-formamide group of glutamine in proteins, which ultimately results in the formation of macromolecular protein cross-linking products [8]. It has been shown that TGase-induced G-L cross-links exhibit low hydrolysis/enzymatic efficiency in organisms; however, G-L cross-links can be absorbed and transported by the intestinal epithelium, and are subsequently cleaved by shear cleavage by γ-glutamyl cyclotransferase and γ-glutamyl transpeptidase, resulting in the release of the free amino acid and no longer substituting the ε-amino acid of the glutamine, so that the TGase and the TGase-induced protein products of its isopeptide bonds do not appear to affect protein utilization in organisms [9,10].

Nowadays, 7S gels are widely used in the food industry as substitutes for meat products, dairy alternatives, baked goods, functional foods, and frozen foods, significantly improving the texture and taste of products to meet consumer demands for healthy and diverse food options [11,12]. As an increasing number of people consume soy 7S protein gel products, it becomes particularly important to study their effects on the human body. These gels not only provide a rich source of plant protein and nutrients but may also have positive effects on digestion, immunity, and metabolism. Understanding the specific impacts of 7S gels on the human body helps to prevent potential risks, ensure food safety, and develop products with greater health benefits. In this study, the 7S gel formed by TGase induced under different ultrasonic pretreatment times was used as the research object to observe the growth characteristics, intestinal morphology, and the change rule of intestinal bacterial flora in mice, and to clarify the effect of the 7S gel on the healthy mice from the biological point of view, with a view to providing theoretical references for the safety of 7S gel applications in the food industry.

## 2. Materials and Methods

### 2.1. Materials

The specific pathogen-free-grade male C57BL/6 mice (*n* = 36, 4–5 weeks old) were purchased from the Center for Comparative Medicine, Yangzhou University. The room temperature was maintained at (23 ± 2) °C with 50% humidity and a 12:12 light–dark cycle. The experimental animal feed was laboratory-standard purified feed AIN-93G (Jiangsu Xietong Pharmaceutical Bio-engineering Co., Ltd., Nanjing, Jiangsu, China) purchased from the Center for Comparative Medicine, Yangzhou University. The β-conglycinin gel samples were prepared based on previous experimental results [1].

### 2.2. Mice Care

The experiment was divided into three phases, i.e., the adaptive feeding phase, intervention phase, and sampling phase.

Adaptive feeding phase: All mice were fed with standard chow for 1 week of acclimatization.

Intervention phase (5 weeks): At the end of the acclimatization feeding stage, the mice were randomly divided into the normal control group (NC, *n* = 6) and gel intervention groups according to the principle of similarity in body weight. Thereafter, the gel intervention groups were randomized into five subgroups: HIU pretreated 0 min gel group (0-gel group, *n* = 6), HIU pretreated 15 min gel group (15-gel group, *n* = 6), HIU pretreated 30 min gel group (30-gel group, *n* = 6), HIU pretreated 45 min gel group (45-gel group, *n* = 6), and HIU pretreated 60 min gel group (60-gel group, *n* = 6). The NC group was gavaged with 0.2 mL of deionized water per day and the 0-gel, 15-gel, 30-gel, 45-gel, and 60-gel groups were gavaged with 0.2 mL/d of gel (gel concentration of 10%). Mice were allowed to eat and drink freely during the gavage period. The body weight, food intake, and water intake of mice were recorded each week, and the feces of the mice in the last week of the intervention experiment were collected.

Sampling phase: The mice were fasted for 24 h, but water was allowed before sampling began, in order to empty the gastrointestinal tract of residual food. Subsequently, the mice were killed by removing the eyeballs, and a portion of the small intestinal tissue was removed and fixed in a centrifuge tube containing 4% paraformaldehyde solution.

The mice used in the study were approved by the Experimental Animal Ethics Committee of Yangzhou University. All experimental conditions and procedures followed the Guide for the Care and Use of Experimental Animals issued by the Experimental Animal Ethics Committee of Yangzhou University.

### 2.3. Small Intestine Sections Observation

After the mouse small intestine segments (jejunum) were fixed by 4% paraformaldehyde for 48 h, the small intestine tissue blocks (15 mm × 15 mm × 5 mm) were taken for tissue section staining experiments with the following steps:

(1) Washing: rinsing with running water for 24 h.
(2) Dehydration: gradient dehydration with anhydrous ethanol.
(3) Transparency: the dehydrated tissue block was immersed in xylene until transparency was achieved.
(4) Wax dipping: dip the tissue blocks into melted paraffin wax: paraffin I (melting point 50–52 °C, 1 h), paraffin II (melting point 52–54 °C, 1 h), paraffin III (melting point 54–56 °C, 1 h).
(5) Embedding: after dipping the wax, placed the paraffin wax into the embedding frame, removed the embedding frame after the paraffin wax cooled and solidified, and trim the wax into a trapezoidal shape for use.
(6) Sectioning: cut thin slices of tissue with a thickness of 4 μm (transverse sections).
(7) Staining, dehydration, and transparency: Hematoxylin-eosin (HE) staining, gradient anhydrous ethanol-xylene dehydration to transparency, sealing with a drop of neutral resin, drying, and then observing the changes in the small intestine tissue sections under a light microscope.
(8) Analysis: The length of the mouse villi was determined using Image-J v1.8.0 image analysis software.

### 2.4. Fecal Sample Collection

Lift up the back of the neck by pinching the mouse and fixing the tail (gavage position). After defecation, the fecal samples were immediately collected into sterile freezing tubes and snap-frozen with liquid nitrogen. The fecal samples were stored in the refrigerator at −80 °C before testing. It is worth noting that prolonged lifting of the back of the mouse’s neck should be avoided to prevent asphyxiation.

### 2.5. Intestinal Flora Testing

The fecal samples were transported to Shanghai Meiji Biomedical Technology Co., Ltd. (Shanghai, China) on dry ice for DNA extraction of intestinal flora, 16S rRNA gene amplification, and sequencing detection on Illumina Miseq PE300 platform. (Shanghai Meiji Bio-Medical Technology Co., Ltd.) Bioinformatic processing of the sequences was performed on the Meiji BioSignal Cloud Platform (https://cloud.majorbio.com). The specific methods are as follows: Quality control and filtering of sequencing sequences: The sequencing sequences were quality controlled and filtered using fastp v0.23.4 software to obtain sequences that meet the requirements. Operational Taxonomic Unit (OTU) Clustering Analysis: OTU clustering of sequencing sequences was performed using Uparse software (v7.0.1090) with a similarity threshold of 97%, followed by rarefaction curve analysis and diversity analysis. Rank–Abundance Curve and Principal Co-ordinates Analysis (PCoA): The analysis software and algorithms used were R software (v3.3.1) and Qiime software (v1.9.1). COG and KEGG Functional Enrichment Analysis: PICRUSt software (v1.1.0).

### 2.6. Statistical Analysis

All measurements were performed in triplicate, and results were expressed as mean ± standard deviation (SD). Data were analyzed by analysis of variance (ANOVA) using SPSS version 19.0 (SPSS Institute, Chicago, IL, USA) and the separation of means was tested by Duncan’s multiple range test at a significance level of *p* < 0.05.

## 3. Results and Discussion

### 3.1. Effects of Gels on Body Weight, Food Intake, and Water Intake in Mice

The effect of the ingested gel on the health of the mice as well as the digestion and absorption of the mice was reflected by recording the differences in body weight gain, food intake, and water intake. The changes in indices of the mice throughout the experimental period are shown in Table 1. The body weight of mice was increased in all groups, indicating that mice grew after ingestion of gels prepared with different HIU pretreatment times. Compared with the NC group, the mice in the 30-gel, 45-gel, and 60-gel groups showed elevated body weight increments, especially in the 30-gel group, where the maximum body weight increment was achieved. It indicated that the 30 min HIU pretreated gel could increase the growth level of mice most dramatically. The food and water intake of the mice in all six groups were not significantly different from each other (*p* > 0.05). However, among the gel intervention groups, the food and water intake of the 15-gel and 30-gel groups were higher than those of the NC group, with the highest increment in the 30-gel group.

### 3.2. Effects of Gels on Intestinal Morphology

**Figure 1:**
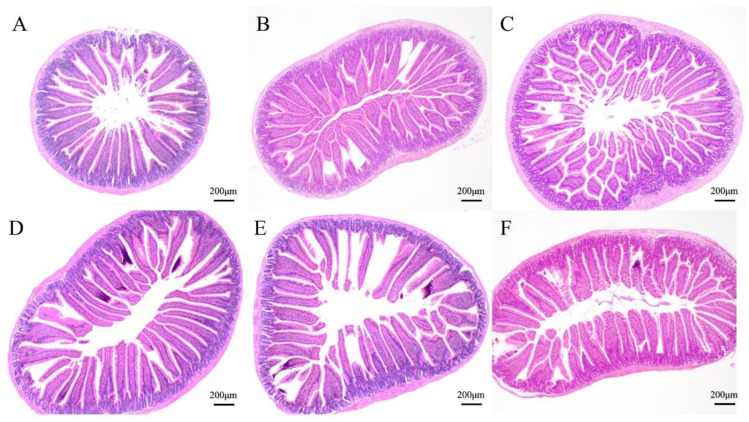
Analysis of morphological changes in the small intestine of mice. (**A**) NC, normal control group; (**B**) 0-gel, HIU pretreated 0 min gel group; (**C**) 15-gel, HIU pretreated 15 min gel group; (**D**) 30-gel, HIU pretreated 30 min gel group; (**E**) 45-gel, HIU pretreated 45 min gel group; (**F**) 60-gel, HIU pretreated 60 min gel group.

Changes in the intestinal morphology of the mice were recorded under the light microscope. As shown in Table 1, compared with the NC group, the length of intestinal villi increased in all gel intervention groups. The changes were significant in the 15-gel, 30-gel, and 45-gel groups, reaching (515.06 ± 26.99) μm, (599.27 ± 44.28) μm, and (571.23 ± 48.77) μm, respectively (*p* < 0.05). In the gel intervention groups, the length of intestinal villi showed a trend of increasing and then decreasing, reaching the longest length significantly in the 30-gel group (*p* < 0.05). The histological sections (Figure 1) showed that the intestinal tissues in the gel intervention group showed better integrity, especially in the 30-gel group, in which the intestinal villi were the most elongated and their arrangement was the most compact and neatly intact. The above results indicated that changes in the duration of HIU treatment significantly affected the length of intestinal villi and changes in gut morphology, and 30-min HIU pretreated gel can help to improve the intestinal morphology and increase the contact area of the small intestine with nutrients [13], which can further help to improve the intestinal digestion and absorption of nutrients and water [14,15].

### 3.3. Quality Assurance of Intestinal Flora Samples

**Figure 2:**
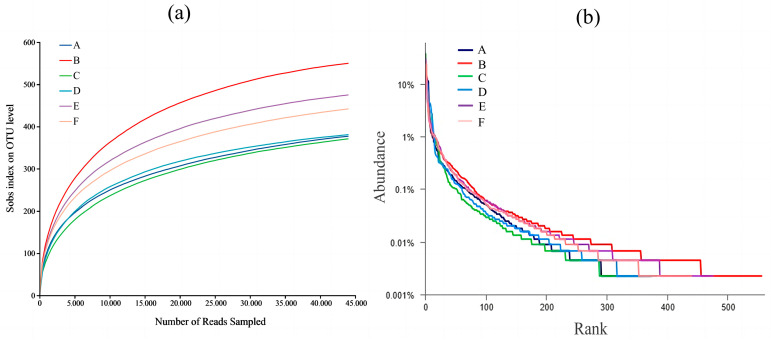
Quality identification of mice intestinal microbiota samples. (**a**) Sob dilution curves; (**b**) Rank–Abundance curves. A, NC group; B, 0-gel group; C, 15-gel group; D, 30-gel group; E, 45-gel group; F, 60-gel group.

In order to judge whether the amount of sequencing data in this experiment is reasonable or not, non-repetitive sequences were identified by OTU division using 97% similarity as a threshold, and Sobs dilution curves and Rank–Abundance curves were constructed. The Sobs dilution curves showed (Figure 2a) that the rise in the number of species detected in the fecal samples increased dramatically as the depth of sequencing gradually deepened when the amount of sequencing data was small. At a cumulative amount of 15,000 sequencing data, the Sobs curves for the six sets of experimental samples gradually flattened out. The above results indicate that the amount of sequencing data in this experiment is reasonable and the sequencing depth is sufficient. The Rank–Abundance rank curve (Figure 2b) reflects the level of species abundance and species evenness, where species richness is proportional to the range of the curve on the horizontal axis; species evenness is proportional to the smoothness of the curve. As the width in the horizontal direction deepens, the range of the curve on the horizontal axis gradually becomes larger, and the curve takes on a flat shape (indicating smoothness), which indicates that species abundance and evenness are relatively high [16]. In conclusion, the sequencing depth of the six groups of samples in this experiment is sufficient and reasonable, and it can reflect the information of most species in each group. In addition, the richness and homogeneity of the microorganisms in the mouse gut flora were good.

### 3.4. Changes in the Diversity of Gut Flora

#### 3.4.1. Alpha-Diversity Analysis

Alpha-diversity analysis reflects the diversity of species in a single sample, and the main indices analyzed include the Chao1 index, Ace (abundance-based coverage estimators) index, Shannon index, and Simpson diversity index. The Ace index (assessing the number of community OTUs) and Chao1 (estimating the total number of species) reflect colony abundance, and the magnitude of both values is proportional to colony abundance. The Shannon index (estimation of community diversity in samples) and Simpson index (quantification of community diversity in a given area) reflect community diversity and are proportional and inversely proportional to community diversity, respectively. Compared with the NC, the intake of gel resulted in the improvement of the abundance and diversity of the intestinal flora of mice (Table 2). With the increase in ultrasound time, the change in the Ace, Chao1, Shannon, and Simpson indices of the gel intervention group was not significant, indicating that the change in HIU pretreatment time did not have a significant effect on the abundance and diversity of the intestinal flora of the mice, and that the homogeneity of the diversity of the intestinal flora of the mice was similar in all groups.

#### 3.4.2. Beta-Diversity Analysis

**Figure 3:**
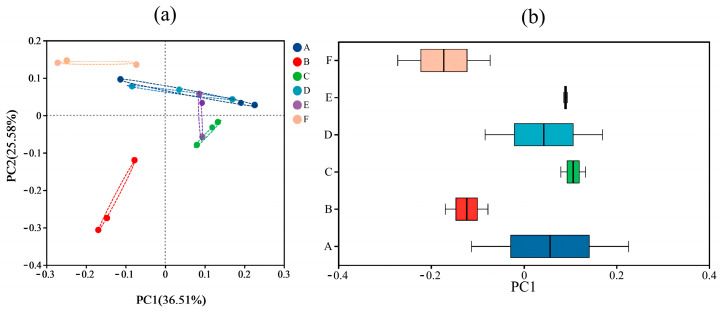
Intergroup analysis of PCoA based on Weighted Unifrac distance (**a**) and distribution dispersion on the PC1 axis (**b**). A, NC group; B, 0-gel group; C, 15-gel group; D, 30-gel group; E, 45-gel group; F, 60-gel group.

**Figure 4:**
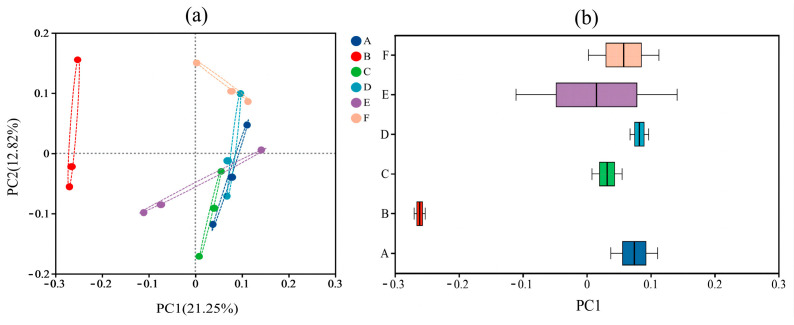
Intergroup analysis of PCoA based on Unweighted Unifrac distance (**a**) and distribution dispersion on the PC1 axis (**b**). A, NC group; B, 0-gel group; C, 15-gel group; D, 30-gel group; E, 45-gel group; F, 60-gel group.

Between-group differences in the composition of the gut microbial communities of different samples were explored using β-diversity analysis. In this study, between-group differences in the composition of the gut microbial communities of six groups of samples were analyzed at the OTU level based on Weighted Unifrac Distance and Unweighted Unifrac Distance based on Principal Co-ordinates Analysis (PCoA), and the results are shown in Figure 3 and Figure 4.

The PCoA plot reflected the similarity of the species composition of the gut microbiota in the samples, and the different colored dots in the plot indicated the samples from different groups; the closer the distance between the dots was, the greater the similarity and the lesser the difference; conversely, the greater the spacing was, the lesser the similarity and the greater the difference. The analysis found that in the Weighted Unifrac distance-based PCoA, the intestinal flora samples from different groups of mice were each clustered, indicating that the structure of the intestinal microbial community in the groups was well clustered. The distance between the samples of the 30-gel group and the NC group was more similar, and both of them were distributed in the first and the second quadrants; the distance between the other gel intervention groups and the NC group was farther away (Figure 3a). The above results indicate that the change in ultrasonication time causes significant changes in the structure of the mouse intestinal microbiota. The PCoA box plots represent the discrete distribution of different groups of samples on the PC1 axis, and the results are shown in Figure 3b. Compared with the samples from the NC group, the samples from the gel intervention group had significantly shorter distribution distances of the gut microbiota, and the cluster distribution was relatively concentrated, suggesting that the intake of gel improved the structural composition of the intestinal flora in mice. The results of PCoA analysis based on Unweighted Unifrac distances showed (Figure 4a) that the distances of the gel intervention groups (except for the 0-gel group) were similar to those of the NC group, which was related to the subtle changes in the intestinal flora of each group. In Figure 4b, the *p*-value for all experimental groups was less than 0.05, indicating that the change in the time of HIU pretreatment resulted in a significant effect on the β-diversity of the mice at the Unweighted Unifrac-based distance.

### 3.5. Analysis of the Composition of Gut Flora

**Figure 5:**
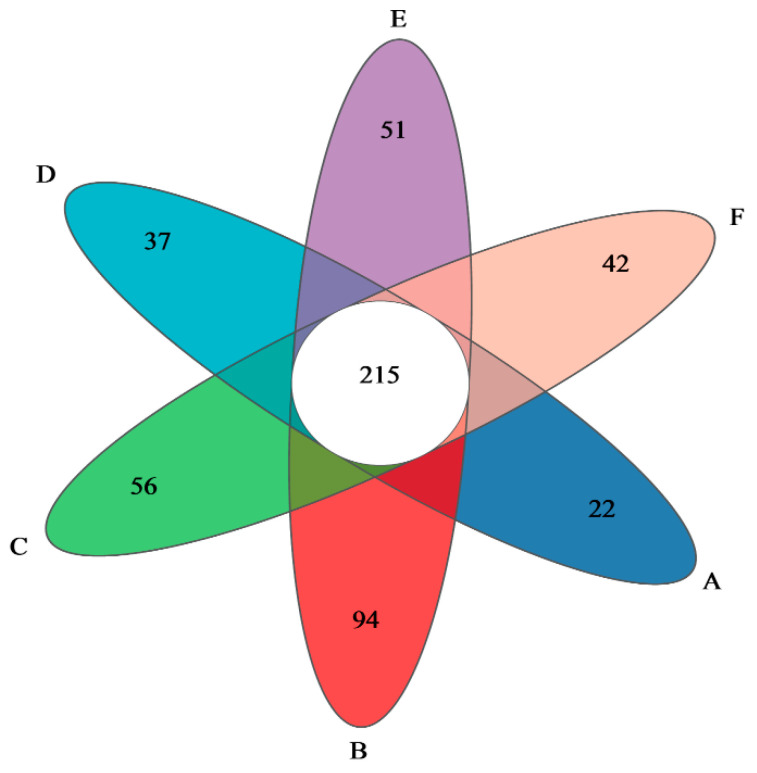
Venn diagram of OTU. A, NC group; B, 0-gel group; C, 15-gel group; D, 30-gel group; E, 45-gel group; F, 60-gel group.

The Venn diagram obtained from the clustering analysis of OTUs can be used to count the number of species shared by different experimental groups of mice and unique to individual samples, which is helpful to show the overlap and similarity of the species composition in the intestinal flora samples of six groups of mice, and the results of the analysis are shown in Figure 5. There were 215 identical OTUs in the six groups of mice; 22 OTUs were unique to the NC group, and 94, 56, 37, 51, and 42 OTUs were unique to the 0-gel, 15-gel, 30-gel, 45-gel, and 60-gel groups, respectively, which were all increased compared to the NC group. Thus, gel ingestion increased the abundance of intestinal flora in mice, and different ultrasound pretreatment times resulted in differences in intestinal flora abundance.

**Figure 6:**
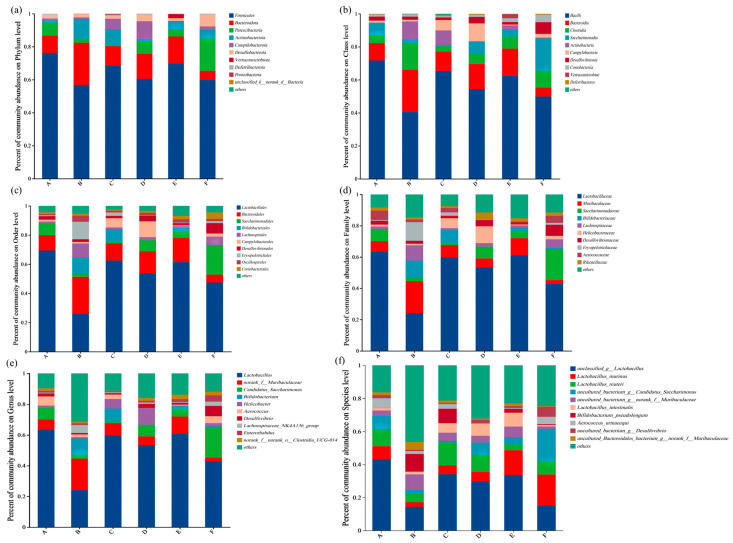
Histogram of community structure distribution of mice gut microbiota at phylum (**a**), class (**b**), order (**c**), family (**d**), genus (**e**), and species (**f**) levels. A, NC group; B, 0-gel group; C, 15-gel group; D, 30-gel group; E, 45-gel group; F, 60-gel group.

To further obtain species taxonomic information corresponding to OTU, the representative sequences of OTU were taxonomically analyzed at 97% similarity level, and the species composition of the microbial communities of each group of samples was counted at different taxonomic levels (Phylum, Class, Order, Family, Genus, and Species), as shown in Figure 6.

At the Phylum level (Figure 6a), the dominant phyla in all six groups of samples were *Firmicutes*, *Bacteroidetes*, *Patescibacteria*, *Actinobacteria*, *Campilobacterota*, *Desulfobacterota*, *Verrucomicrobia*, *Deferribacterota*, and *Proteobacteria*, with the six phyla with the highest relative abundance (*Firmicutes*, *Bacteroidetes*, *Patescibacteria*, *Actinobacteria*, *Campilobacterota,* and *Desulfobacterota*) containing more than 95% of the flora sequences in each set of samples. Moreover, the relative abundance of the dominant flora was all somewhat altered by gel ingestion.

At the Class level (Figure 6b), the six groups of samples were divided into a total of 10 classes: *Bacilli*, *Bacteroidia*, *Clostridia*, *Saccharimonadia*, *Actinobacteria*, *Campylobacteria*, *Desulfovibrionia*, *Coriobacteriia*, *Verrucomicrobacteria*, and *Deferribacteres*. The first eight of these accounted for 95% of the relative abundance of each group of samples’ sequences of more than one group of bacteria.

At the Order level (Figure 6c), the taxonomic units of each group of samples were categorized into 10 orders: *Lactobacillales*, *Bacteroidales*, *Saccharimonadales*, *Bifidobacteriales*, *Lachnospirales*, *Campylobacteriales*, *Desulfovibrionales*, *Erysipiplobacteriales*, *Oscillospirales*, and *Coriobacteriales*.

At the Family level (Figure 6d), the taxonomic units of each group of samples were categorized into 10 families: *Lactobacillaceae*, *Muribaculaceae*, *Saccharimonadaceae*, *Bifidobacteriaceae*, *Lachnospiraceae*, *Helicobacteraceae*, *Desulfovibrionaceae*, *Erysipelotrichaceae*, *Aerococcaceae*, and *Rikenellaceae*.

At the Genus level (Figure 6e), the taxonomic unit was divided into 10 genera, namely *Lactobacillus*, *norank_f_Muribaculaceae*, *Candidatus_Saccharimonas*, *Bifidobacterium*, *Helicobacter*, *Aerococcus*, *Desulfovibrio*, *Lachnospiraceae_NK4A136_group*, *Enterorhabdus*, and *norank_f_norank_o_Clostridia_UCG-014*.

At the Species level (Figure 6f), taxonomic units were categorized into 10 mediums: *unclassified_g_Lactobacillus*, *Lactobacillus_murinus*, *Lactobacillus_reuteri*, *uncultured_bacterium_g_Candidatus_Saccharimonas*, *uncultured_bacterium_g_norank_f_Muribaculaceae*, *Lactobacillus_intestinalis*, *Bifidobacterium_pseudolongum*, *Aerococcus_urinaeequi*, *uncultured_bacterium_g_Desulfovibrio*, and *uncultured_Bacteroidales_bacterium_g_norank_f_Muribaculaceae.*

After ultrasonic treatment, the abundance of *Bacilli* and *Lactobacillales* increased to varying degrees (Figure 6b), and the abundance of *Bifidobacteriales* significantly increased in the 0-gel and 15-gel groups (Figure 6c). These bacteria may help maintain the balance of the gut microbiota, increase the digestibility of nutrients, promote intestinal health [17,18,19], or have potential protective effects in slowing the progression of certain diseases [19,20].

Compared to the NC, the abundance of *Firmicutes* and *norank_f_Muribaculaceae* in the gut microbiota of mice decreased after gel intervention, while the abundance of *Actinobacteria* and *Lactobacillus* increased (Figure 6a,e). This can promote the production of primary bile acids to some extent, maintain the homeostasis of the bile acid pool, and accelerate the conversion of liver cholesterol to bile acids [21]; the increased abundance of *Actinobacteria* also helps enhance the biotransformation of bile acids (BAS) [22], thereby reducing the risk of fat accumulation caused by a high-fat diet.

Except for the 60-gel group, the abundance of *Bacteroidetes* in all gel intervention groups increased compared to the untreated group (Figure 6a), which may help reduce the risk of non-alcoholic fatty liver disease (NAFLD) to some extent [23]. Additionally, the increased ratio of *Bacteroidetes* to *Firmicutes* may help reduce the occurrence of gut inflammation and chronic diseases such as obesity and diabetes [24].

These results indicate that the composition of the gut microbiota in mice changes after gel intake intervention, which in turn affects the overall health of the mice. Additionally, the variation in HIU pretreatment time has a significant impact on the changes in bacterial community structure. Changes in bacterial groups such as *Firmicutes* may affect the intestinal health and blood lipid levels of the mice.

### 3.6. Prediction of Biological Functions of Intestinal Flora

PICRUSt functional prediction software was used to analyze all 16S sequencing data (COG analysis and KEGG analysis) to predict the functional gene composition of each group of samples, and thus to analyze the major biological functions involved in the different subgroups as well as the functional differences between them [25].

#### 3.6.1. COG Biofunction Prediction

**Figure 7:**
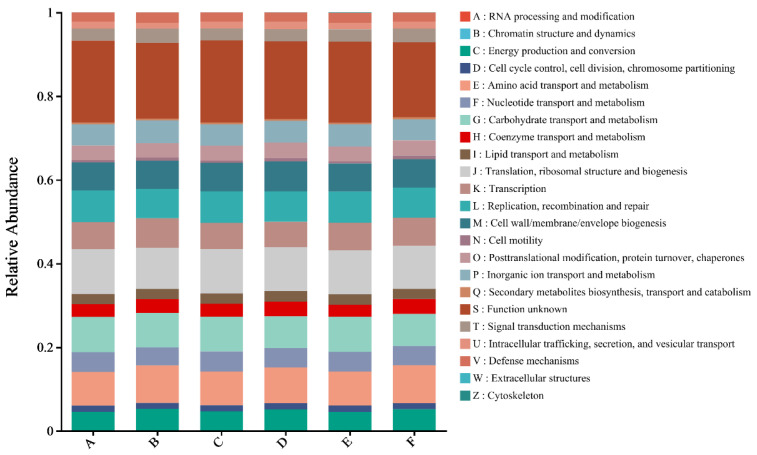
Functional relative abundance bar chart based on COG analysis. A, NC group; B, 0-gel group; C, 15-gel group; D, 30-gel group; E, 45-gel group; F, 60-gel group.

The results of functional classification and relative abundance analysis of COG are shown in Figure 7. Compared with the normal NC, gel intake up-regulated the COG function of mouse gut microbial community in A: RNA processing and modification, C: Energy production and conversion, E: Amino acid transport and metabolism, M: Cell wall/membrane/envelope biogenesis, P: Inorganic ion transport and metabolism, T: Signal transduction mechanisms, and V: Defense mechanisms, which were up-regulated. The expression of genes related to G: Carbohydrate transport and metabolism was down-regulated. Compared with the group without ultrasonically pretreated gel (0-gel group), the intestinal flora COG function was down-regulated in the areas of D: Cell cycle control, cell division, and chromosome partitioning; F: Nucleotide transport and metabolism; I: Lipid transport and metabolism; J: Translation, ribosomal structure, and biogenesis; L: Replication, recombination, and repair; M: Cell wall/membrane/envelope biogenesis; O: Posttranslational modification, protein turnover, chaperone; and U: Intracellular trafficking, secretion, and vesicular transport, which were up-regulated. The abundance of C: Energy production and conversion, E: Amino acid transport and metabolism, K: Transcription, P: Inorganic ion transport and metabolism, and V: Defense mechanisms were down-regulated. In the 30-gel group, the abundance of D: Cell cycle control, cell division, and chromosome partitioning; O: Posttranslational modification, protein turnover, and chaperones; and U: Intracellular trafficking, secretion, and vesicular transport functions were at their highest abundance.

The above results indicate that gel intake can contribute to the improvement of intestinal health in mice by altering the physiological function of the intestinal flora, and that gels prepared with different HIU pretreatment times produced significant differences in the alteration of the physiological function of the intestinal flora.

#### 3.6.2. Prediction of KEGG Biological Function

To further explore the functional changes of the mouse gut microbial community after the intervention of gels prepared with different HIU pretreatment, all sequencing data were analyzed using KEGG analysis to predict primary functional pathways, secondary subfunctional pathways, and tertiary metabolic pathways.

##### KEGG Tier One Functional Prediction

**Figure 8:**
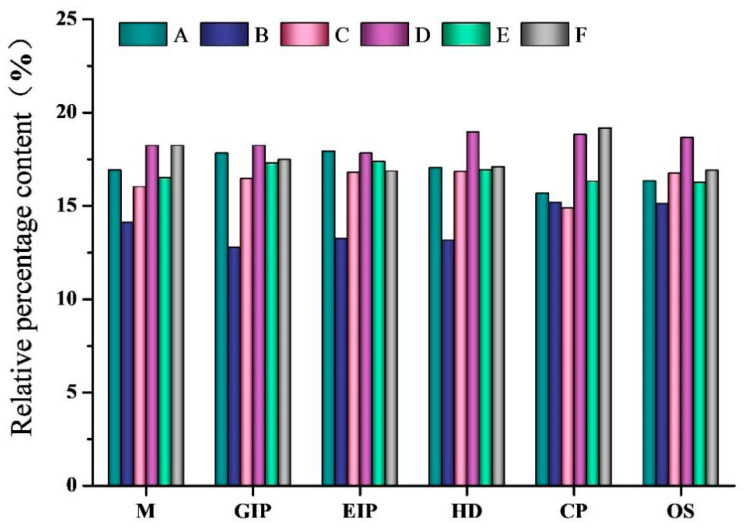
Functional relative abundance bar chart based on first level annotation of KEGG analysis. M: Metabolism; GIP: Genetic Information Processing; EIP: Environmental Information Processing; HD: Human Diseases; CP: Cellular Processes; OS: Organismal Systems. A, NC group; B, 0-gel group; C, 15-gel group; D, 30-gel group; E, 45-gel group; F, 60-gel group.

The results of the comparative analysis of the relative abundance of biological functions predicted by the first tier of functions are shown in Figure 8. This database contains six major biometabolic pathways: Metabolism, Genetic Information Processing, Environmental Information Processing, Human Diseases, Cellular Processes, and Organismal Systems. The relative abundance of genetic information processing function was the highest in the NC, 15-gel, 30-gel, 45-gel, and 60-gel groups, and the highest relative abundance in the 0-gel group was metabolism, while the other predicted functional pathways were basically the same. Gel ingestion reduced the relative abundance of intestinal flora in the metabolic pathways of human diseases compared to the NC group. The KEGG-annotated predictive functions were significantly reduced after ingestion of gels prepared without HIU pretreatment (0-gel group); on the contrary, the KEGG predictive functions of the mouse intestinal flora gradually returned to normal when ingesting gels prepared with HIU pretreatment, and the relative abundance of the predictive functions of metabolism, genetic information processing, and organismal systems was highest in the 30-gel group.

##### Analysis of KEGG Second-Level Subfunctional Pathways

**Figure 9:**
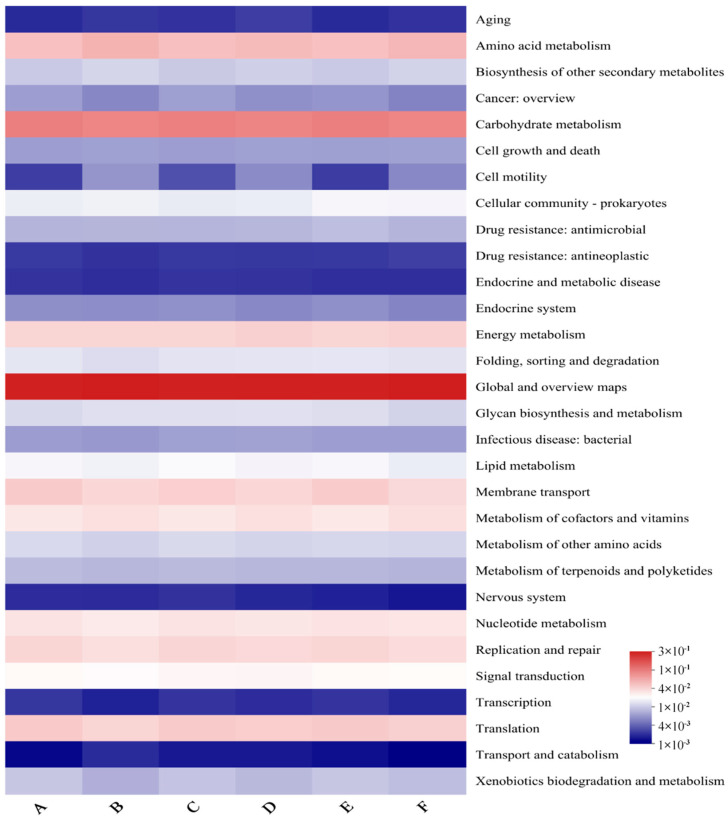
KEGG heat map. A, NC group; B, 0-gel group; C, 15-gel group; D, 30-gel group; E, 45-gel group; F, 60-gel group.

Further correlation heatmap analysis of the predicted second-tier subfunctional pathways was performed, and the results are shown in Figure 9. The top ten predicted pathways in terms of abundance were Global and overview maps, Carbohydrate metabolism, Amino acid metabolism, Translation, Membrane transport, Energy metabolism, Replication and repair, Metabolism of cofactors and vitamins, Nucleotide metabolism, and Signal transduction, which mainly involves a series of functional pathways such as energy metabolism, amino acid metabolism, and transmembrane transport. In order to better reflect the similarities and differences among the six groups of KEGG second-level functional prediction results, correlation heatmaps were drawn for the top thirty functional pathways in relative abundance (Figure 9). It can be clearly seen that there is less variability in the KEGG functional prediction results among the groups.

##### Analysis of KEGG Tertiary Metabolic Pathways

In the final search for KEGG tertiary metabolic pathways, a total of 290 pathways were annotated in this experiment, and the pathways with the top 30 clustering numbers in the clustering difference were compared, and the results are shown in Table 3. Compared with the NC group, gel intake down-regulated the genes of metabolic pathways in ko02010 (ABC transporters), ko00010 (Glycolysis/Gluconeogenesis), ko00520 (Amino sugar and nucleotide sugar metabolism), ko00500 (Starch and sucrose metabolism), ko02060 (Phosphotransferase system), and ko00052 (Galactose metabolism). The number and proportion of genes clustered in the top 30 pathways were significantly up-regulated after ingestion of the HIU pretreated gel compared with the 0-gel group.

Additionally, compared to the other five experimental groups, the 30-gel group had the highest number of metabolic pathway genes in ko01100 (Metabolic pathways), ko03010 (Ribosome), ko01200 (Carbon metabolism), ko02020 (Two-component system), ko00240 (Pyrimidine metabolism), ko00970 (Aminoacyl-tRNA biosynthesis), ko00620 (Pyruvate metabolism), ko03440 (Homologous recombination), ko00550 (Peptidoglycan biosynthesis), ko00250 (Alanine, aspartate and glutamate metabolism), ko03430 (Mismatch repair), ko00190 (Oxidative phosphorylation), ko00680 (Methane metabolism), ko03030 (DNA replication), and ko00720 (Carbon fixation pathways in prokaryotes).

The abundance of metabolic pathway genes in the gut microbiota enables it to break down and utilize various dietary components, including complex carbohydrates, proteins, and fats. This not only helps the host absorb nutrients more efficiently but also produces beneficial metabolic products, such as short-chain fatty acids (SCFAs) and bile acids, which can maintain immune homeostasis in the gut and throughout the body, playing an important role in the host’s health [26,27].

## 4. Conclusions

In this study, TGase-induced 7S gels prepared at different HIU pretreatment times (0, 15, 30, 45, and 60 min) were selected for intervention in healthy mice. The results showed that the body weight of mice increased in all groups (30-gel > 60-gel > 45-gel > NC > 0-gel). There was no significant difference (*p* > 0.05) in food intake and water intake among the six experimental groups of mice; however, the 30-gel group had the maximum average daily food intake and water intake. Compared with the NC group, the intake of gel increased the length of small intestinal villi in mice, with the highest value of villi length in the 30-gel group (*p* < 0.05). HE staining of small intestinal tissue sections showed that the intestinal tissues of the six groups of mice presented better integrity, indicating that the gel did not adversely affect the intestinal tract, and that the intestinal villi were the longest and presented the neatest and tightest arrangement in the 30-gel group. The change in the pretreatment time of the HIU did not have a significant effect on the alpha-diversity of the intestinal flora of the mice (*p* > 0.05), and the abundance and diversity of the flora were similar; the effect on the β-diversity of intestinal microbial community structure was more significant (*p* < 0.05). Analysis of the composition of the intestinal flora showed that the OTU of the intestinal flora of mice in the gel intervention group were all increased compared with that of the NC group. COG and KEGG analyses revealed that gel intake could improve the intestinal health of mice by altering the physiological function of the intestinal flora, and that the gels prepared with different HIU pretreatment times had a significant effect on the alteration of the physiological function of the intestinal flora. In addition, the predictive functions of KEGG annotations were significantly reduced in the 0-gel group; on the contrary, the KEGG predictive functions of the intestinal flora of mice gradually returned to normal when ingesting gels prepared with ultrasonication pretreatment, and the relative abundance of the predictive functions of metabolism, genetic information processing, and organismal systems reached the highest in the 30-gel group; this indicates that ultrasonication pretreatment significantly enhances the beneficial effects of 7S gels on intestinal health.

## Figures and Tables

**Table 1:** Analysis of changes in body weight gain, food intake, water intake, and length of intestinal villi in mice of different treatment groups.

Index	Group					
	NC	0-Gel	15-Gel	30-Gel	45-Gel	60-Gel
Weight gain (g/mouse)	3.60 ± 0.82 ^abc^	2.98 ± 1.04 ^bc^	1.92 ± 1.19 ^c^	5.30 ± 0.93 ^a^	4.02 ± 1.78 ^ab^	4.51 ± 0.81 ^ab^
Food intake (g/day/mouse)	4.48 ± 0.68 ^a^	4.63 ± 0.46 ^a^	4.95 ± 0.71 ^a^	5.20 ± 1.32 ^a^	4.77 ± 0.10 ^a^	5.04 ± 0.62 ^a^
Water intake (g/day/mouse)	7.38 ± 1.66 ^a^	6.90 ± 2.68 ^a^	7.90 ± 2.43 ^a^	8.33 ± 2.80 ^a^	6.89 ± 1.40 ^a^	7.00 ± 1.89 ^a^
Intestinal villus length (μm)	428.73 ± 38.03 ^c^	454.29 ± 72.30 ^c^	515.06 ± 26.99 ^b^	599.27 ± 44.28 ^a^	571.23 ± 48.77 ^a^	434.67 ± 25.26 ^c^

Note: Values are expressed as mean ± standard deviation. Means with different letters in the same line are significantly different at *p* < 0.05, while those with the same letters are not significantly different. NC, normal control group, gavaged with 0.2 mL deionized water. The groups were as follows: 0-gel, HIU pretreated 0 min gel group, gavaged with 0.2 mL/d of gel (gel concentration of 10%); 15-gel, HIU pretreated 15 min gel group, gavaged with 0.2 mL/d of gel (gel concentration of 10%); 30-gel, HIU pretreated 30 min gel group, gavaged with 0.2 mL/d of gel (gel concentration of 10%); 45-gel, HIU pretreated 45 min gel group, gavaged with 0.2 mL/d of gel (gel concentration of 10%); 60-gel, HIU pretreated 60 min gel group, gavaged with 0.2 mL/d of gel (gel concentration of 10%).

**Table 2:** Alpha-diversity index of intestinal flora of mice in different treatment groups.

Index	Groups					
	NC	0-Gel	15-Gel	30-Gel	45-Gel	60-Gel
Ace	421.70 ± 45.49 ^a^	664.48 ± 79.90 ^a^	437.29 ± 8.51 ^a^	431.18 ± 31.12 ^a^	464.14 ± 97.91 ^a^	467.66 ± 72.30 ^a^
Chao1	405.50 ± 43.26 ^a^	641.11 ± 78.73 ^a^	424.13 ± 12.47 ^a^	412.18 ± 29.96 ^a^	448.54 ± 99.82 ^a^	456.28 ± 63.74 ^a^
Shannon	2.41 ± 0.94 ^a^	3.83 ± 0.56 ^a^	2.79 ± 0.03 ^a^	2.94 ± 0.34 ^a^	2.86 ± 0.60 ^a^	2.94 ± 0.38 ^a^
Simpson	0.28 ± 0.25 ^a^	0.07 ± 0.04 ^a^	0.16 ± 0.01 ^a^	0.14 ± 0.08 ^a^	0.17 ± 0.07 ^a^	0.13 ± 0.01 ^a^

Note: Values are expressed as mean ± standard deviation. Means with different letters in the same line are significantly different at *p* < 0.05, while those with the same letters are not significantly different.

**Table 3:** Comparison of the number of clusters of KEGG metabolic pathways in the intestinal flora of mice in different treatment groups.

Metabolic	Groups					
Pathway	NC	0-Gel	15-Gel	30-Gel	45-Gel	60-Gel
ko01100	23,439,746	19,360,321	22,336,993	25,354,327	22,925,673	24,892,139
ko01110	10,456,317	9,254,657	9,910,042	11,610,032	10,212,205	11,975,884
ko01120	5,779,879	4,734,169	5,491,556	6,186,953	5,589,684	6,268,392
ko01230	4,132,845	4,211,400	3,910,724	4,821,597	4,104,280	5,313,082
ko03010	4,091,703	2,904,285	3,773,697	4,171,639	3,953,634	3,987,781
ko01200	3,632,795	2,968,635	3,397,004	3,928,264	3,529,986	3,967,398
ko02010	3,386,694	2,750,944	3,190,401	3,285,163	3,350,084	3,264,017
ko00230	2,513,030	1,883,457	2,389,565	2,567,332	2,453,262	2,669,402
ko02020	2,348,392	1,940,732	2,354,091	2,674,206	2,322,834	2,419,457
ko00010	2,398,224	1,580,678	2,169,217	2,247,200	2,311,216	2,252,075
ko00520	2,269,628	1,570,781	2,084,154	2,216,457	2,268,899	2,013,616
ko00500	2,294,951	1,551,009	2,070,266	1,943,892	2,247,550	1,823,264
ko00240	2,017,036	1,448,176	1,909,123	2,033,974	1,956,324	1,921,928
ko02024	1,855,968	1,507,906	1,688,810	1,877,558	2,023,999	2,036,021
ko00970	1,871,519	1,341,629	1,740,058	1,927,406	1,820,459	1,847,727
ko00620	1,581,537	1,180,863	1,514,130	1,672,443	1,504,536	1,616,424
ko03440	1,516,975	1,117,059	1,402,726	1,543,021	1,492,658	1,504,727
ko02060	1,801,536	871,704.1	1,491,779	1,325,477	1,644,406	1,191,803
ko00550	1,408,416	1,053,988	1,302,987	1,402,598	1,363,430	1,404,262
ko00250	1,308,220	1,093,091	1,258,017	1,392,205	1,264,763	1,338,927
ko00052	1,443,285	990,006.1	1,335,246	1,309,394	1,398,118	1,166,213
ko03430	1,344,278	962,723.4	1,235,516	1,370,198	1,302,062	1,321,198
ko00270	1,176,242	1,093,446	1,176,320	1,287,636	1,176,006	1,368,999
ko00190	1,213,440	956,018.2	1,162,266	1,435,505	1,178,794	1,302,796
ko00680	1,274,113	930,364.4	1,173,188	1,324,706	1,240,242	1,300,340
ko00260	1,184,458	1,001,140	1,100,288	1,235,962	1,159,757	1,346,824
ko00030	1,223,938	969,190	1,129,966	1,214,481	1,173,019	1,237,497
ko00051	1,302,862	880,650.4	1,159,914	1,200,922	1,286,395	1,068,187
ko03030	1,139,027	810,182.4	1,059,922	1,165,906	1,103,110	1,118,433
ko00720	1,044,721	888,098.8	978,078.5	1,238,024	1,028,684	1,202,155

Note: ko01100: Metabolic pathways; ko01110: Biosynthesis of secondary metabolites; ko01120: Microbial metabolism in diverse environments; ko01230: Biosynthesis of amino acids; ko03010: Ribosome; ko01200: Carbon metabolism; ko02010: ABC transporters; ko00230: Purine metabolism; ko02020: Two-component system; ko00010: Glycolysis/Gluconeogenesis; ko00520: Amino sugar and nucleotide sugar metabolism; ko00500: Starch and sucrose metabolism; ko00240: Pyrimidine metabolism: ko02024: Quorum sensing; ko00970: Aminoacyl-tRNA biosynthesis; ko00620: Pyruvate metabolism; ko03440: Homologous recombination; ko02060: Phosphotransferase system; ko00550: Peptidoglycan biosynthesis; ko00250: Alanine, aspartate, and glutamate metabolism; ko00052: Galactose metabolism; ko03430: Mismatch repair; ko00270: Cysteine and methionine metabolism; ko00190: Oxidative phosphorylation; ko00680: Methane metabolism; ko00260: Glycine, serine, and threonine metabolism; ko00030: Pentose phosphate pathway; ko00051: Fructose and mannose metabolism; ko03030: DNA replication; ko00720: Carbon fixation pathways in prokaryotes.

## Data Availability

The original contributions presented in the study are included in the article, further inquiries can be directed to the corresponding author.
